# Synergism of a mixed diet of *Myzus persicae* and egg of *Ephestia kuehniella* on fitness of the predator *Nabis stenoferus*

**DOI:** 10.1038/s41598-023-35363-6

**Published:** 2023-06-05

**Authors:** Young-gyun Park, Minhyeok Kwon, Souvic Sarker, Un Taek Lim

**Affiliations:** 1 Department of Plant Medicals, Andong National University, Andong, Republic of Korea; 2 Present Address: Department of Entomology, Purdue University, West Lafayette, IN USA; 3 Present Address: Department of Entomology, Rutgers, The State University of New Jersey, New Brunswick, NJ USA

**Keywords:** Population dynamics, Entomology

## Abstract

*Nabis stenoferus* is a zoophytophagous predator that lives in grasslands around agricultural fields. It is a candidate biological control agent for use via augmentation or conservation. To find a suitable food source for mass-rearing and to better understand this predator’s biology, we compared the life history characteristics of *N. stenoferus* under the three different diets: aphids only (*Myzus persicae*), moth eggs only (*Ephestia kuehniella*), or a mixed diet of aphids and moth eggs. Interestingly, when only aphids were supplied, *N. stenoferus* developed to the adult stage but lacked normal levels of fecundity. There was a significant synergism of the mixed diet on *N. stenoferus* fitness in both the immature and adult stages, i.e., a 13% reduction in the nymphal developmental period and an 87.3-fold increase in fecundity, compared to aphid-only diet. Furthermore, the intrinsic rate of increase was significantly higher for the mixed diet (0.139) than either aphids only (0.022) or moth eggs only (0.097). These results show that *M. persicae* alone is not a complete diet for the mass-rearing of *N. stenoferus,* whereas this aphid can be a supplementary food when combined with *E. kuehniella* eggs. Implications and applications of these findings for biological control are discussed.

## Introduction

The zoophytophagous predator *Nabis stenoferus* Hsiao (Hemiptera: Nabidae) is a natural enemy of prey such as aphids, moths, and spider mites in Korea, Japan, and China^1–3^. *Nabis stenoferus* can also be a pest in some crop plants, such as chrysanthemum [*Chrysanthemum* spp. (Asterales: Asteraceae)] due to its zoophytophagous habit^1^. Generally, this predator lives in grasslands around agricultural fields, and it has been regarded principally as a biodiversity component of agroecosystems rather than a pest or biocontrol agent^2–4^. However, *N. stenoferus* has the potential to be a new biological control agent for use in augmentation or conservation programs because of its generalist feeding habit and adaptation to temperate regions^3,4^. Generalist predators can be especially useful as biological control agents in crops where several pest species occur at the same time^5^. Also, a generalist may be better able to survive under low pest densities than a specialist as alternative food sources are available to them^5–7^.

Efficient mass-rearing of natural enemies is essential if agents are to be used in augmentative biocontrol^8^. For efficient mass-rearing, it is crucial to find suitable environmental conditions such as temperature and relative humidity^9–11^, however, adequate diet is also a critical factor^12,13^. The food item used for insect mass-rearing should be inexpensive, easily obtained, and suitable for both the species’ development and oviposition^14^.

Also, even though a particular food may not be part of the insect’s natural diet, such foods may be used for rearing insects if they provide sufficient nutrients^14^. Eggs of stored product moths such as *Ephestia kuehniella* Zeller (Lepidoptera: Pyralidae) are frequently used for mass-rearing generalist predators^15,16^. This moth is a pest of stored grain and is not found in Korea, but its frozen eggs have been imported into Korea for the mass-rearing of insect predators^17,18^. However, it can be argued that foods that are part of a species’ diet under natural conditions might be more effective than foods never yet encountered by a generalist predator^19^. *Myzus persicae* (Sulzer) (Hemiptera: Aphididae) is a common agricultural pest in crops in the families Asteraceae, Brassicaceae, Cucurbitaceae, and Solanaceae^20–23^. This aphid is found in open fields in agricultural areas in Korea, Japan, and China, and its host plants and habitats overlap with those of *N. stenoferus*^1,3,20^. Also, this aphid has sometimes been used as a supplementary food for the rearing of generalist predators^24^.

Life table analysis allows the population parameters of insects under specific conditions to be calculated. Among life table parameters, the intrinsic rate of increase is a comprehensive and intuitive parameter describing the population potential of an insect reared under specific conditions that can be calculated from data on developmental time, fecundity, longevity, sex ratio, and survivorship^25,26^. Population parameters derived from life table analysis can be statistically compared using bootstrap or jackknife methods^25,27^. Thus, life table analysis is a very effective tool for comparing the fitness of insects under different conditions. However, life table analysis of *N. stenoferus* has rarely been done.

Our goal in this study was to find a suitable diet for mass-rearing *N. stenoferus* and to better understand its biology. We compared its life history characteristics using life table analysis for groups reared using three diets: (1) aphids only (*M. persicae*), (2) moth eggs only (*E. kuehniella*), and (3) a mixed diet of aphids and moth eggs.

## Results

### Immature stages

There were no significant differences among the three treatments (aphids only, eggs only, or both) in the development periods of the egg to 3rd nymph stages (Table 1) (egg, *χ*^2^ = 0.13, *df* = 2, *P* = 0.935; 1st nymph, *χ*^2^ = 3.70, *df* = 2, *P* = 0.157; 2nd nymph, *χ*^2^ = 5.51, *df* = 2, *P* = 0.064; 3rd nymph, *χ*^2^ = 4.53, *df* = 2, *P* = 0.104). However, the developmental periods of the 4th and 5th nymphal instars, as well as that of the total immature period for groups fed a mixed diet were significantly shorter than in either the aphid-only or moth egg-only diets (Table 1) (4th nymph, *χ*^2^ = 17.78, *df* = 2, *P* < 0.001; 5th nymph, *χ*^2^ = 27.45, *df* = 2, *P* < 0.001; total immature, *χ*^2^ = 31.50, *df* = 2, *P* < 0.001).

**Table 1:** Developmental periods (days, mean ± SE) of immature stages of *Nabis stenoferus* fed three different diets. *Means followed by the same letter within a column are not significantly different at α = 0.05. DSCF multiple comparisons as a post hoc test for the Kruskal–Wallis test.

Diet	Egg (n)	1st nymph (n)	2nd nymph (n)	3rd nymph (n)	4th nymph (n)	5th nymph (n)	Total immature (n)
Aphids	8.4 ± 0.11a* (20)	2.6 ± 0.11a (20)	2.1 ± 0.16a (20)	2.4 ± 0.21a (20)	3.4 ± 0.13a (20)	5.9 ± 0.25a (19)	24.8 ± 0.56a (19)
Moth eggs	8.5 ± 0.11a (20)	2.9 ± 0.11a (20)	2.2 ± 0.16a (20)	2.3 ± 0.14a (20)	3.2 ± 0.08a (20)	5.4 ± 0.19a (19)	24.3 ± 0.29a (19)
Aphids + Moth eggs	8.5 ± 0.11a (20)	2.6 ± 0.11a (20)	1.8 ± 0.10a (20)	2.0 ± 0.00a (20)	2.6 ± 0.11b (20)	4.2 ± 0.09b (20)	21.6 ± 0.23b (20)

### Adult females

There were significant differences among the diet treatments in the life history characteristics of adult females of *N. stenoferus* (Table 2). The preoviposition period was significantly shorter in the group fed a mixed diet than in aphid-only or moth egg-only group (*F* = 16.11, *df* = 2, 17; *P* < 0.001). The oviposition periods varied from 5.7 to 41.6 days among the treatments, but without statistical significance (*χ*^2^ = 5.85, *df* = 2, *P* = 0.054). The postoviposition period in the group fed the aphid-only diet was significantly longer than in moth egg-only group (1.4 days) (*χ*^2^ = 7.50, *df* = 2, *P* = 0.024). There was no significant difference in female adult longevity among the diets tested (*F* = 1.76, *df* = 2, 25; *P* = 0.193). The total fecundity per female of bugs fed the mixed diet (aphids + moth eggs) was significantly higher than in groups fed the other two diets (*F* = 14.75, *df* = 2, 25; *P* < 0.001).

**Table 2:** Oviposition period, longevity, and fecundity (mean ± SE) of *Nabis stenoferus* reared from eggs on three different diets. *Means followed by the same letter within a column are not significantly different at α = 0.05. Tukey’s studentized range test as a post hoc test for analysis of variance or DSCF multiple comparisons as a post hoc test for the Kruskal–Wallis test.

Diet	Preoviposition period (day) (n)	Oviposition period (day) (n)	Postoviposition period (day) (n)	Longevity (day) (n)	Total fecundity (eggs/female) (n)
Aphids	13.0 ± 3.06a* (3)	5.7 ± 1.76a (3)	10.0 ± 2.65a (3)	26.3 ± 6.02a (7)	5.7 ± 2.82b (7)
Moth eggs	7.9 ± 0.64b (8)	37.4 ± 6.26a (8)	1.4 ± 0.18b (8)	37.6 ± 7.02a (11)	206.8 ± 52.37b (11)
Aphids + Moth eggs	4.4 ± 0.41c (9)	41.6 ± 3.47a (9)	3.4 ± 1.61ab (9)	44.9 ± 5.95a (10)	497.7 ± 80.74a (10)

### Life table parameters by treatment

**Figure 1:**
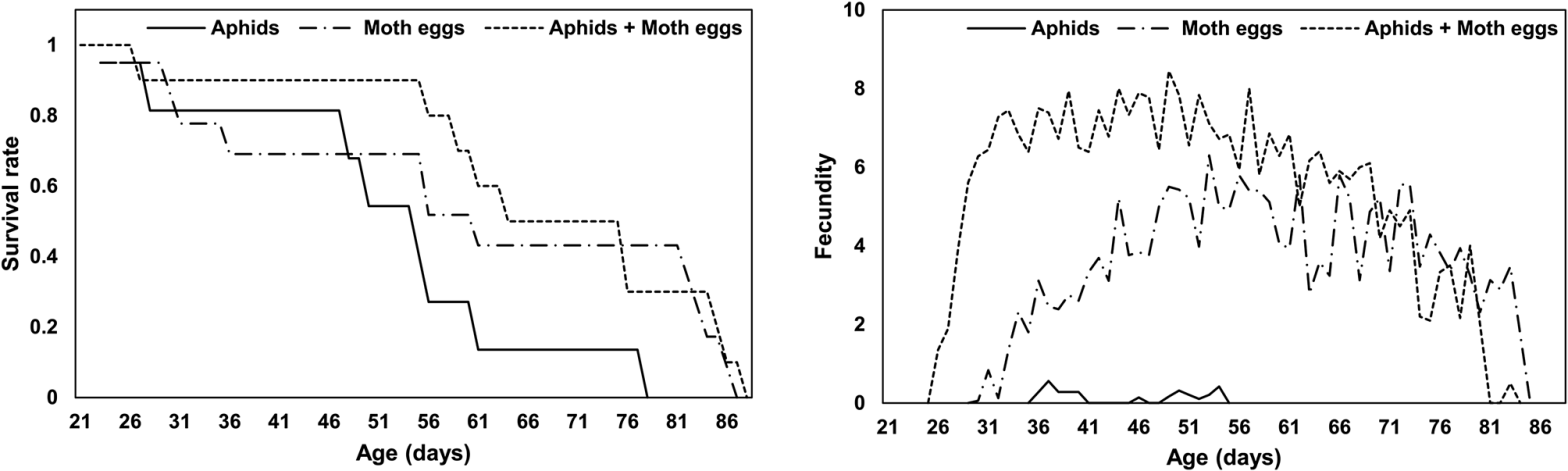
Age-specific survival rate (*l*_*x*_) and fecundity (*m*_*x*_) curves of *Nabis stenoferus* fed three different diets.

The age-specific survival rate of *N. stenoferus* for nymphs fed the aphid-only diet decreased more rapidly than did rates of groups fed the other diet (Fig. 1). Fecundity was also much lower in the group fed the aphid-only diet (Fig. 1). Different diet treatments significantly affected the population parameters of *N. stenoferus* (Table 3). The intrinsic rate of increase and finite rate of increase were highest in insects fed the mixed diet, followed by the moth egg-only group and the aphid-only group. The net reproductive rate also was highest in insects fed the mixed diet. Furthermore, the net reproductive rate in the group fed the aphid-only diet was relatively lower compared to insects on the mixed diet, but was not significantly different compared to moth eggs only diet. There was no significance among treatments in the mean generation time.

**Table 3:** Population parameters (estimate ± SE) of *Nabis stenoferus* fed three different diets. *Means followed by the same letter within a column are not significantly different at α = 0.05, Tukey’s studentized range test after jackknife estimates.

Diet	Net reproductive rate (${R}_{0}$)	Mean generation time ($T$)	Intrinsic rate of increase (${r}_{m}$)	Finite rate of increase ($\lambda$)
Aphids	2.286 ± 1.1270b*	42.921 ± 5.5849a	0.022 ± 0.0123c	1.023 ± 0.0125c
Moth eggs	113.750 ± 28.8046b	48.846 ± 2.2674a	0.097 ± 0.0063b	1.102 ± 0.0069b
Aphids + Moth eggs	248.850 ± 40.3720a	39.884 ± 0.6824a	0.139 ± 0.0044a	1.149 ± 0.0050a

## Discussion

In this study, we found significant effects of different food sources on the life history characteristics of *N. stenoferus*. Moth eggs alone did provide sufficient nutrients for both insect development and adult oviposition by *N. stenoferus*, but use of the aphid-only diet yielded sterile, or nearly sterile, adults. The mean fecundity in insects reared on the mixed diet was 87 times higher than bugs reared on the aphid-only diet. However, the fecundity of bugs reared on the moth egg diet was not significantly different from the group reared on aphid-only diet probably due to the very low number of ovipositing females obtained. Furthermore, the aphid-only diet lowered adult fitness of *N. stenoferus* by prolonging both the pre- and post-oviposition periods. Aphids are known to be low-quality foods for generalist predators, likely due to the presence of toxins or feeding deterrents^24^. However, in our study, there were no negative effects of an aphid-only diet on the development of the immature stage of *N. stenoferus*, and, consequently, we surmise that low adult fertility might be due to a nutritional deficiency in the aphid-only diet. Both protein and lipid diet components are known to significantly affect insect fecundity^28–30^. Proteins and lipids comprise 51.0 and 33.6% of the dry mass of *Ephestia* eggs^31^. In contrast, aphid-only diets have a lower lipid content than *Ephestia* eggs^28–30^. Different dietary needs for development in the immature stage versus reproduction in the adult stage are well known^32,33^. Different food exploitation patterns between immature and adult stages have frequently been reported in predatory mites. For example, immature stages of *Phytoseiulus persimilis* Athias‐Henriot (Acari: Phytoseiidae) can consume thrips and complete their stage’s development despite a lower survival rate of this food^34^. However, the adult *P. persimilis* was known to rarely consume thrips as food^34^. Thus, a *M. persicae*-only diet might be suitable for the development of *N. stenoferus* nymphs but not suitable for adult maturation is consistent with other systems.

However, there was a synergistic effect of aphid and moth egg diets for the life history characteristics of *N. stenoferus* in both the immature and adult stages. Numerous studies have reported the advantages of a mixed diet on insect fitness^35–37^. However, the noteworthy finding in our study is that the diet on which *N. stenoferus* completed its nymphal development (but yielded sterile adults) had a synergistic effect when mixed with other diet. Toft et al.^30^, in which *Ephestia* eggs and/or *Rhopalosiphum padi* (L.) aphids were used as food for *Orius majusculus* Reuter (Hemiptera: Anthocoridae), found lower fecundity in the aphid diet than in *Ephestia* egg diet. However, unlike our study, there was no synergistic effect on *O. majusculus*’ fecundity from a mixed diet. In contrast, we found that the fecundity of *N. stenoferus* fed a mixed diet of aphids and moth eggs more than doubled compared to the moth eggs-only diet. The developmental period of the immature stage and the preoviposition period of *N. stenoferus* were also significantly shorter for bugs reared on the mixed diet. Furthermore, the mixed diet resulted in insects with a higher intrinsic rate of increase than did the other two treatments. This is the first finding of such contrasting contributions of diets on the development and reproduction of Hemiptera as far as we know.

Zoophytophagous hemipteran predators facultatively consume plant sap to obtain nutrients and water, but they can complete their life cycles only by feeding on animal prey such as insects or mites, as shown for *N. stenoferus* in this study^38–42^. However, some zoophytophagous predators can complete their life cycles by feeding only on plants without prey. For example, *Nesidiocoris tenuis* (Reuter) (Hemiptera: Miridae) can successfully increase its population by feeding only on sesame plants [*Sesamum indicum* L. (Lamiales: Pedaliaceae)]^43^, although it was not able to do so when feeding only on tomato plants [*Solanum lycopersicum* L. (Solanales: Solanaceae)]^44^. The *Ephestia* egg diet did not show synergistic effects on the fitness of *N. tenuis* when paired with a tomato plant^39^. However, *Ephestia* egg diet did have synergistic effects on development periods and fecundity of *N. tenuis* when paired with a sesame plant^43^, showing that plant-derived components from a suitable host plant could positively affect the fitness of zoophytophagous predators^45^. In our study, we supplied Chinese cabbage [*Brassica rapa* L. subsp. *pekinensis* (Brassicales: Brassicaceae)] leaves to *N. stenoferus* as its water source and oviposition substrate. Chinese cabbage might not be able to increase the population size of *N. stenoferus* without prey because the aphid-only diet in our study did not show good adult fitness. However, the nutrients derived from Chinese cabbage might be converted and enriched in the aphids, and these nutrients in a mixed diet might enhance the fitness of *N. stenoferus*. Thus, in our study, *M. persicae* in a mixed diet with eggs of *E. kuehniella* might have played a similar role for *N. stenoferus* as a beneficial plant did for *N. tenuis* in the previous example.

Even though *M. persicae* alone is not a complete food source for *N. stenoferus*, it might be an error to regard this predator as an ineffective biological control agent against *M. persicae* or other aphid species. A diet that improves a predator's fitness does not necessarily engender a higher preference for that diet compared to others, even ones with intrinsically poorer nutrient profiles^30^. In Toft et al.^30^, *O. majusculus*, when reared on *Ephestia* eggs only or on a mixed diet of eggs and aphids, still preferred the poorer quality aphid-only diet. However, this predator, when reared on an aphid-only diet showed no preference between *Ephestia* eggs and aphids. Even though a diet might not be able to provide all the nutrients a predator might need, if the diet has the essential nutrients such as vitamins and amino acids, the predator may still consume the diet to prevent nutrient deficiency^46^. Unlike laboratory conditions, where the type of diet is artificially restricted, under natural conditions predators have opportunities to exploit mixed host resources efficiently for the best fitness gain^46^. The natural prey of *N. stenoferus* are known to be moth eggs or larvae, spider mites, and aphids^3^; and *N. stenoferus* should maximize its fitness by exploiting mixed diets in nature. However, in agricultural areas, especially in greenhouses, a predator’s diet choices may be limited by the simplification of the agroecosystem^47,48^. In predator-based augmentative biological control programs against aphid species in greenhouses, supplemental provision of *Ephestia* eggs might improve control^49^. Also, it may be possible to plant chrysanthemums in greenhouses as banker plants for *N. stenoferus* because this predator can complete its life cycle on chrysanthemums alone^1^. However, in open grassy fields, where *N. stenoferus* is an indigenous predator^2,3^, the species would be appropriate for conservation biological control^50,51^. Building up refuges with chrysanthemum plants can be suitable to enhance this predator than probably food spraying of *Ephestia* eggs, which can be used by other antagonists such as ants protecting aphids^52,53^.

In conclusion, the overall fitness of *N. stenoferus* was higher on a diet of *E. kuehniella* eggs than a diet of *M. persicae*, and a mixed diet of *M. persicae* and *E. kuehniella* eggs had a synergistic effect on the fitness of *N. stenoferus* in both the immature and adult stages. Therefore, we propose that *M. persicae* can be a supplementary food source for the mass-rearing of *N. stenoferus*, with the eggs of *E. kuehniella* the primary food source. Further studies might be needed on the nutritional composition of both *E. kuehniella* eggs and *M. persicae* to identify essential nutrients that might be responsible for the better fitness of *N. stenoferus*. To establish an efficient mass-rearing system for *N. stenoferus*, studies on the effects of environmental conditions such as temperature and humidity on *N. stenoferus* would also be needed. Moreover, as the use of *Ephestia* eggs for mass breeding of *N. stenoferus* may become expensive in certain regions or under specific conditions, additional research might be necessary to investigate the feasibility of utilizing relatively inexpensive diets such as brine shrimp eggs^54^.

## Methods

### Food sources

The eggs of *E. kuehniella* were used to feed our laboratory colony of *N. stenoferus*. Combinations of both *E. kuehniella* eggs and *M. persicae* (nymphs and adults mixed) were tested for the diet suitability. Both eggs and aphids were purchased from the Osang Kinsect, Namyangju, Korea. The eggs of *E. kuehniella* were frozen in the bottles received and taken out and used whenever necessary. Seedlings (about 10 cm height) of Chinese cabbage (*B. rapa* subsp. *pekinensis*) were purchased from the market in Andong, Korea, and planted in each pot (10 cm × 9.7 cm; diameter × height) and kept at 27 ℃, 60–80% RH, and a 16:8 (L:D) h photoperiod in an incubator (DS-50CPL, Dasol Scientific Co., ltd., Suwon, Korea) until plants grow to 20 cm. *Myzus persicae* stages were reared in an acrylic cage (30 × 30 × 30 cm) on three pots of Chinese cabbage at 27 ℃, 60–80% RH, and a 16:8 (L:D) h photoperiod in an incubator. Chinese cabbage pots were replaced at two to three week intervals. All methods were carried out in accordance with the relevant guidelines and regulations of Republic of Korea.

### Laboratory rearing of *Nabis stenoferus*

*Nabis stenoferus* was obtained from the Gyeonggi-do Agricultural Research and Extension Service in Hwaseong, Korea, and bugs were reared individually from egg to adult in Petri dishes (35 mm × 10 mm; diameter × height; SPL Life Science, Pocheon, Korea) at 27 ℃, 60–80% RH, and a 16:8 (L:D) h photoperiod in an incubator. Eggs of *E. kuehniella*, attached on parchment paper (1 × 1 cm), were provided as food for *N. stenoferus* rearing, and the egg papers were replaced daily. A piece of water-saturated cotton (0.8 × 0.8 × 0.8 cm) was placed in the rearing Petri dishes to supply water and as an oviposition substrate. When *N. stenoferus* individuals became adults, a pair of predators were kept in the Petri dish for mating.

### Life table experiments

The experiment on rearing diets was conducted at 27.9 ± 0.76 ℃, 50.1 ± 6.4% RH, and a 16:8 (L:D) h photoperiod in an incubator. Twenty individuals (as newly laid eggs) of *N. stenoferus* were used for each food treatment. To obtain newly laid *N. stenoferus* eggs, ten adult females were randomly collected from the rearing colony for each treatment. Each female was allowed to lay eggs on water-saturated cotton in a Petri dish (35 mm × 10 mm; diameter × height) for 24 h. After 24 h, each female’s cotton pieces were collected into a larger Petri dish (90 mm × 15 mm; diameter × height; SPL Life Science) and held for egg hatch. The eggs developmental period was recorded.

The newly emerged nymphs were randomly selected and placed individually into experimental Petri dishes (50 mm × 15 mm; diameter × height; SPL Life Science) containing water-saturated cotton and a Chinese cabbage leaf disc (50 mm diameter). Diets were provided in Petri dishes as three treatments: (1) aphids only, (2) moth eggs only, and (3) both aphids and moth eggs. Aphid numbers in each Petri dish with aphids were maintained at least 40 individuals daily. The daily supply of aphids might be sufficient for *Nabis* sp. to meet its feeding needs (about 14.4 aphids were consumed per day in a previous study^55^) with no observable food shortage. For treatments with moth eggs, one parchment paper (1 × 1 cm^2^) with several hundred eggs was present in each Petri dish and was replaced daily.

When nymphs molted to adults, individual females and males were paired and held in new experimental Petri dishes. When female could not be immediately paired due to discrepancy in the number of molted males, we used male adults collected from the rearing colony. In the adult stage, only the life history characteristics of females were assessed, according to Maia et al.^25,26^. Developmental times of each life stage, female adult longevity, and daily fecundity were observed daily until all females had died.

### Data analysis

The data from any individuals lost during the experiment were discarded before analysis. Females that did not lay eggs were excluded from the calculation of mean oviposition periods. The developmental periods for nymphs, as well as the oviposition and postoviposition periods were compared among treatments using the Kruskal–Wallis test in PROC NPAR1WAY in SAS^56^ because of the non-normal distribution of the data. The preoviposition periods, as well as female adult longevity and fecundity were compared among treatments by analysis of variance using PROC GLM in SAS^56^.

Life table analysis and jackknife estimation of population parameters were carried out using the R program^57^ by referring to Maia et al.^25,26^. Age-specific survival rates ($${l}_{x}$$) and fecundity ($${m}_{x})$$ for each treatment were calculated using the following equations:

$${l}_{x}=SURV\times \frac{{NSF}_{x}}{NF}$$

$${m}_{x}={NEGG}_{x}\times SR$$

where $SURV$ is the survival rate from egg to adult, ${NSF}_{x}$ is the number of surviving females at age $x$, and $NF$ is the initial number of females. ${NEGG}_{x}$ is the mean number of eggs laid at age $x$, and $SR$ is the sex ratio of each treatment group. The population parameters were calculated by the following equations^25^.

The net reproductive rate (${R}_{0}$)

$${R}_{0}=\sum_{x=0}^{\infty }{l}_{x}{m}_{x}$$

The mean generation time ($T$)

$$T=\frac{\sum_{x=0}^{\infty }x{l}_{x}{m}_{x}}{\sum_{x=0}^{\infty }{l}_{x}{m}_{x}}$$

The intrinsic rate of increase (${r}_{m}$)

$$\sum_{x=0}^{\infty }{e}^{-{r}_{m}x}{l}_{x}{m}_{x}=1$$

The finite rate of increase ($\lambda$)

$$\lambda ={e}^{{r}_{m}}$$

The population parameters such as net reproductive rate (${R}_{0}$), mean generation time ($T$), intrinsic rate of increase (${r}_{m}$), and finite rate of increase ($\lambda$) were compared by Tukey's studentized range test after jackknife estimation^25^.

## Data Availability

The data supporting the findings of this study are publicly available at 10.5281/zenodo.7614004.
